# Accuracy in Furnace Atomic Absorption Spectroscopy

**DOI:** 10.6028/jres.093.113

**Published:** 1988-06-01

**Authors:** W. Slavin

**Affiliations:** Perkin-Elmer Corporation, 901 Ethan Allen Hwy., Ridgefield, CT 06877

This paper holds that, when graphite furnace atomic absorption spectroscopy (GFAAS) is used correctly, it comes as close to being accurate as any analytical technique that has been proposed for trace metal determination. The signal for the analyte does not depend upon the particular matrix. Thus, if GFAAS is used correctly, it should be as close as is presently possible to providing a reference method for trace metals. The same simple standards should suffice for all samples whether they are biologicals, alloys or industrial products.

We admit that there still are situations for which this opportunity does not apply. Sometimes this is because we struggle to make do with outmoded instrumentation that was never designed for the modern graphite furnace or instrumentation that is not working properly. Sometimes it is because some of us don’t want to solve our analytical problem in a simple way. If we try to take a simple and theoretically correct approach, failures of the system to work will help us understand why the theory does not apply.

By now there are more than a hundred papers in the literature confirming situations where the simple and general technique has been used [1], which has come to be called the stabilized temperature platform furnace (STPF). Of course, many more analysts are using these procedures without publishing because they have simply confirmed with reference materials or by comparison with earlier more laborious procedures that the simple methodology worked well.

## Background Correction Error Sources in GFAAS

An important and frequent source of error is the failure of the background correction system to perform its role. Several methods for correcting matrix background errors have been proposed and almost every furnace measurement requires that some kind of background correction be made.

The earliest background correction relied on a measurement with continuum radiation that provided almost no signal for the analyte [2]. This worked very well if the background effects were not too large and if the beams from the two light sources were very carefully superimposed. There are many analytical situations which can be handled well with continuum source background correction but there are more situations which cannot.

In recent years the Zeeman effect has been adapted to background correction [3]. There are several advantages to Zeeman correction but the most important is that only one light source is necessary. This avoids the adjustment problem associated with continuum correction.

Smith and Hieftje [4] suggested that a single source could be used in two modes, the background correction mode being accomplished with an intense self-absorbed pulse from the lamp that is therefore insensitive to the analyte. The major limitation to their proposal, when it is applied to the graphite furnace, is that a considerable time is required after the large lamp pulse for the atomic cloud to dissipate. This delay forces a slow chopping frequency that is incompatible with the fast signals from the furnace.

## Atomization Efficiency

The L’vov theory implies that it is possible to fully atomize the sample, or at least the analyte at the chosen furnace temperature. This was argued for many years but L’vov [5] and others have now shown that for the bulk of the elements determined in the furnace the efficiency of atomization is very close to 100%. Some elements are exceptions to this, and these elements cannot yet be determined in the furnace with confidence that the matrix will not affect the efficiency. These troublesome elements are mainly among the rare earth and some of the alkali and alkaline earth elements. Furnaces that avoid graphite, e.g., by using tantalum [6], may make these elements accessible to interference-free AAS.

Another implication of the requirement for full atomization of the analyte is that the matrix components will not form strong vapor phase bonds that remove the analyte from the atomic state required for AAS. The halides form such vapor phase bonds with many metals and this continues to be troublesome [7]. Matrix modifiers are used to stabilize the analyte so that a char step can be used to remove much of the halide. However, present commercial furnaces have cold ends which condense some portion of the vaporized halide during the char step. This condensed halide is revaporized in the atomization step producing an interference in some situations. Indeed it is this problem that limits the amount of halide that can be present to a few percent for several analytes.

There are several ways to handle this. One is to make a furnace that is free of cold ends [8]. Another way is to avoid the char step entirely and depend upon the halide to have diffused out of the furnace before or after the analyte is vaporized. This usually requires Zeeman correction because the background signals become large when the char step is avoided. We have used this successfully for T1 in the presence of large amounts of chloride but we haven’t studied this approach on a general basis yet.

## Instrumental and Methodological Quality Assurance

Every instrument and every analytical method has the potential for error. Certainly, making available reference materials in a wide variety of matrices, accurately characterized for the many trace metals that must be determined, is an important step towards satisfying this need for quality assurance. We routinely use NBS Standard Reference Material (SRM) 1643 (Trace Metals in Water) as a test of our analytical conditions and of our standards.

This paper has emphasized that the analytical curve from simple standard solutions applies equally to a wide range of matrices. The slope of the linear portion of an analytical curve for each element is reported as characteristic mass, the mass of analyte in pg that produces a 1% absorption (which is 0.0044 absorbance) signal. These are integrated signals so the units are absorbance seconds (A·s). We have shown that instruments with similar furnace geometries provide very similar characteristic masses [9]. Thus, obtaining the expected characteristic mass for a particular analyte is a useful quality assurance indicator.

There are many experimental situations which can alter the apparent characteristic mass and three elements, Ag, Cu and Cr, have been chosen for a test procedure to be used for several purposes. The test confirms that our instrument is working well each day, that different instruments are working similarly and confirms that Zeeman instruments are working well when they leave our factory.

NBS SRM 1643b Trace Metals in Water is used without dilution to avoid pipetting and contamination errors. Instrumental conditions are used which introduce as few variables as possible. No matrix modifier or char step is used. Some of the test data and experience with this test have been reported [10] but much more work must still be done.

While most graphite furnaces are still purchased for routine determination of trace metals in widely varied materials, there is a gradual acceptance of the new furnace technology as the primary means for calibrating standards to be used for other faster but less accurate techniques. There are still instrumental improvements ahead and considerable work is required to simplify the analytical procedures by making fuller use of the new furnace technology.
